# Supplementary material for: Seminoma and Embryonal Carcinoma Footprints Identified by Analysis of Integrated Genome-Wide Epigenetic and Expression Profiles of Germ Cell Cancer Cell Lines
Source: PLoS One. 2014 Jun 2;9(6):e98330. doi: 10.1371/journal.pone.0098330 (PMC4041891; doi:10.1371/journal.pone.0098330)
Supplement: File S1 — ZIP file containing DMRforPairs output for significant regions. Please start from the html files. (ZIP) [file pone.0098330.s008.zip › figures/10304.pdf]

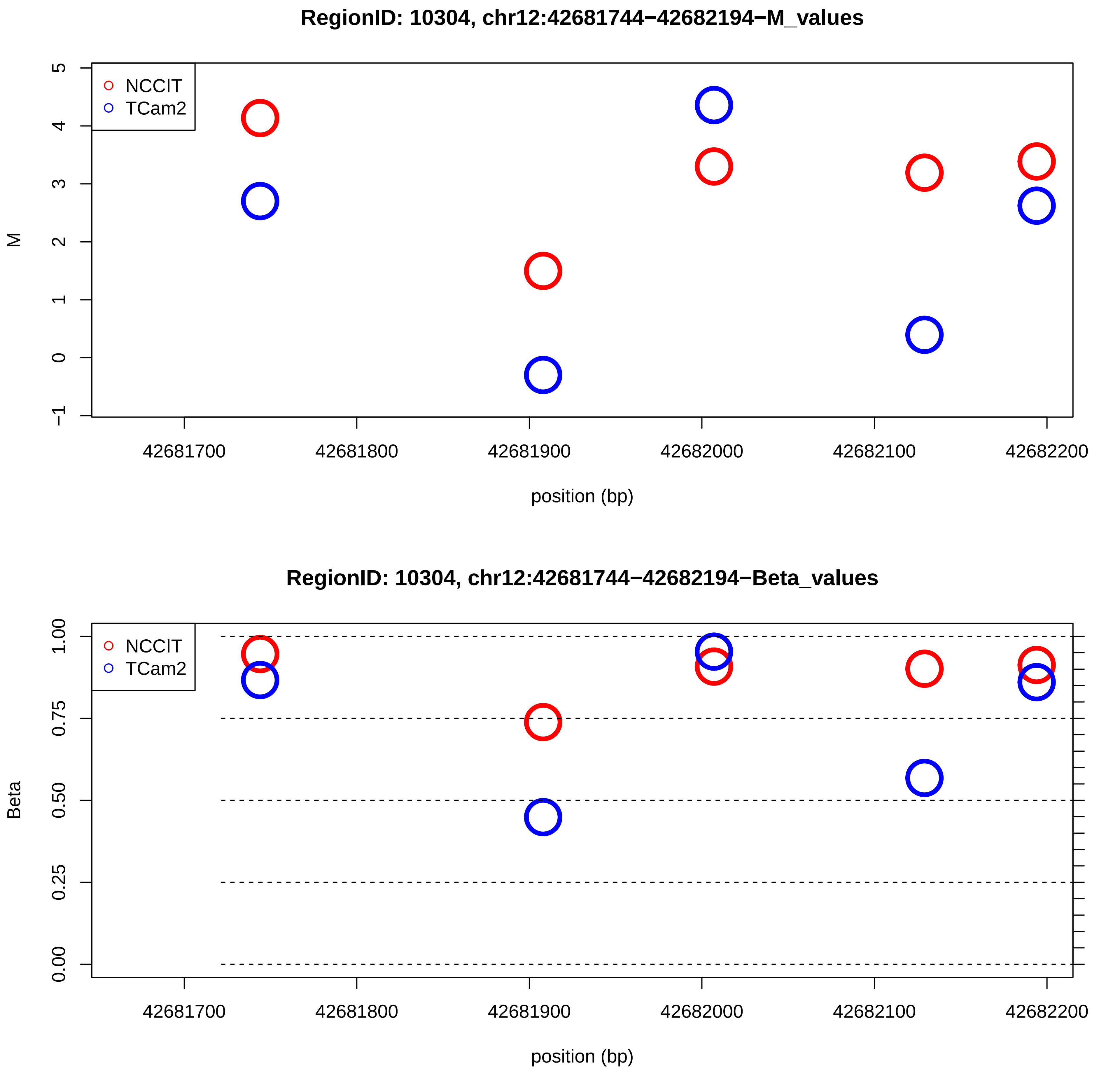
RegionID: 10304, chr12:42681744−42682194−M_values
M
position (bp)
NCCIT
TCam2
−1
0
1
2
3
4
5
42681700
42681800
42681900
42682000
42682100
42682200
RegionID: 10304, chr12:42681744−42682194−Beta_values
Beta
position (bp)
NCCIT
TCam2
0.00
0.25
0.50
0.75
1.00
42681700
42681800
42681900
42682000
42682100
42682200
